# Building research evidence for advancing prevention and translation: reflecting on a 20-year organizational approach of applied chronic disease preventive health research

**DOI:** 10.1093/tbm/ibaf090

**Published:** 2026-01-12

**Authors:** Blythe J O’Hara, Lesley King, Adrian E Bauman, Philayrath Phongsavan

**Affiliations:** Prevention Research Collaboration, Sydney School of Public Health, Faculty of Medicine and Health, The University of Sydney, Camperdown, NSW, 2006, Australia; Charles Perkins Centre, The University of Sydney, Camperdown, NSW, 2006, Australia; Prevention Research Collaboration, Sydney School of Public Health, Faculty of Medicine and Health, The University of Sydney, Camperdown, NSW, 2006, Australia; Charles Perkins Centre, The University of Sydney, Camperdown, NSW, 2006, Australia; Prevention Research Collaboration, Sydney School of Public Health, Faculty of Medicine and Health, The University of Sydney, Camperdown, NSW, 2006, Australia; Charles Perkins Centre, The University of Sydney, Camperdown, NSW, 2006, Australia; Prevention Research Collaboration, Sydney School of Public Health, Faculty of Medicine and Health, The University of Sydney, Camperdown, NSW, 2006, Australia; Charles Perkins Centre, The University of Sydney, Camperdown, NSW, 2006, Australia

**Keywords:** prevention, research translation, health promotion, chronic disease prevention

## Abstract

**Background:**

Translating public health research into practice remains challenging despite ongoing focus on evidence-based approaches. This study profiles the scope of research undertaken by the Prevention Research Collaboration (PRC), a university based applied public health research organization, with funding from both traditional academic sources and from policy agencies, and examines how it contributed to a translational, evidence-building approach in chronic disease prevention.

**Methods:**

We analyzed PRC’s research output using two complementary approaches: (i) a review of journal articles published from 2018 to 2024 where PRC researchers were lead or senior authors; and (ii) an examination of annual reports and workplans from 2013 to 2023 to identify major research programs. Research was classified according to public health evidence-building typology and whether it was investigator-initiated or policy-initiated.

**Results:**

Overall, investigator-initiated research was dominant amongst journal publications, and particularly showcased problem definition studies. Intervention evaluation, as identified in journal publications and internal documents, was more likely to be policy-initiated. PRC demonstrated a high degree of collaboration with policy and practice professionals (42.5% of investigator-initiated and 50% of policy-initiated publications included policy co-authors). Key research areas across chronic disease prevention included physical activity (40.4% of publications), obesity prevention (14.2%), and tobacco control (12.8%).

**Conclusion:**

This case study demonstrates that a small public health research group can successfully navigate the research-policy interface over a sustained period. PRC’s continuity of management, staffing and funding arrangements, plus shared agenda and strong partnership with government, are considered to be key enabling factors for this collaborative evidence-building public health approach.

## Graphical Abstract

**Graphical Abstract:**
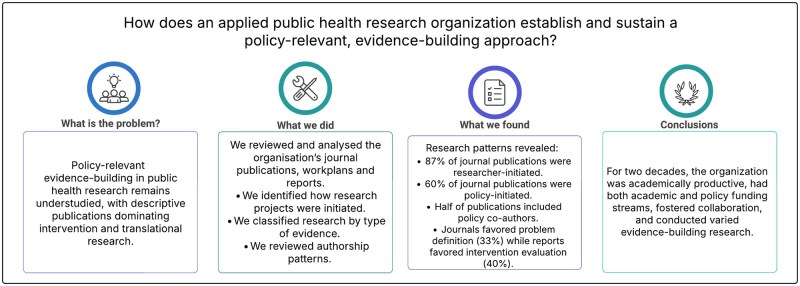

Implications **Practice:** Sustained public health research impact requires deliberate cultivation of long-term collaborative partnerships between researchers and policy professionals, built on shared aims, ongoing communication, and high levels of trust that enable genuine co-production of evidence to inform practice, with the recognition that significant policy-initiated research contributions may not be evident in traditional peer-reviewed publication profiles.

**Policy:** While both policy-initiated and investigator-initiated research can contribute to building evidence for public health, the differences in their focus suggest that there is value in mixed funding sources for research organizations themselves and diverse inclusion of policy/practice stakeholders and interest groups collaborators.

**Research:** Further research beyond a descriptive profile is required to illuminate the ways in which collaborative, policy-oriented public health research can influence policy, practice, and research capacity.

## Introduction

Translating evidence into practice within public health has always been of importance, more so with the resurgence of evidence-based medicine in the 1990s [1]. Translation of research and evidence refers to the processes of linking research findings with real-world policies, practices, and organizational arrangements. Such translation seeks to ensure that policies and practices can improve health and well-being outcomes for populations or vulnerable communities and reduce health inequality [2]. Ongoing interest in evidence-based public health [2, 3] has led to increases in the published literature on research translation and related topics of dissemination, implementation research, and scaling up [4, 5]. Nevertheless, translation remains both a small area of publication, and a rare focus of research grants [6, 7].

**Figure 1:**
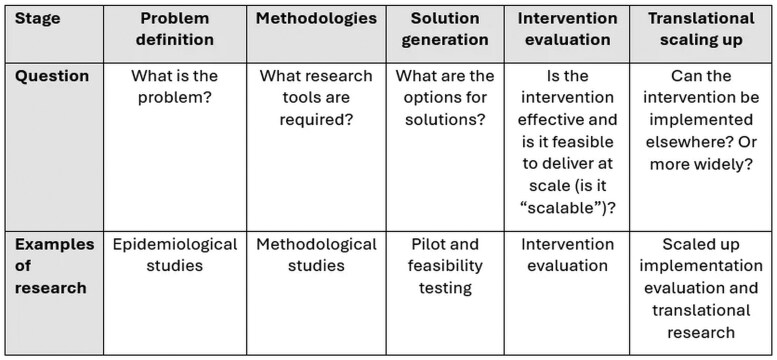
Stages of evidence-building and study types (adapted from Bauman and Nutbeam [8]).

In the first instance, several types of research evidence are required to identify and address public health problems. There are various ways of representing and describing stages of evidence-building and related study types, and they typically cover: problem definition, solution generation, innovative testing/intervention demonstration, intervention translation, and scaling up (as depicted in Figure 1), these stages typically encompass: problem definition (identifying the nature and scope of health issues), methodological development (creating appropriate research tools), solution generation (exploring potential interventions), intervention evaluation (testing effectiveness and scalability), and translational scaling up (implementing interventions in broader contexts) [3, 8]. Each stage employs distinct research approaches—from epidemiological studies and systematic reviews in the problem definition phase, through pilot and feasibility testing during solution generation, to scaled-up implementation evaluation in the translational phase. Thus, a framework distinguishing different types of evidence enables a nuanced view and ways in which research utilization and translation may be analyzed and considered by policy and practice.

Such classification systems recognize that different types of research have different purposes and address different audiences. Thus, a variety of research outputs, measurement tools, evaluation methodologies, and interventions may be the subject of translational efforts. However, in many cases translational efforts are focused on an intervention that has been shown to be effective, seeking for it to be adopted or implemented more widely. The translation stage for an intervention is quite distinctive as it often involves applying the intervention in a new setting and often on a much larger scale or population-wide [9, 10]. Complexities are also introduced as the ‘thing’ being translated can change and may require adaptation when implemented on a large scale or over time [10, 11].

Typologies of evidence-building research have also been used to profile sets of published studies in various topic areas [12–14]. The consistent and unsurprising pattern is that only a small proportion of public health research publications address highly transferable interventions that have been conducted in community and organizational contexts using the existing workforce and infrastructure [14, 15]. There are even fewer reports on scaled-up implementation [9, 14–16]. The processes of translation and scaling up are frequently represented as a series of stages [17]. The traditional linear research translation has been critiqued on the basis that translation is not a unidirectional process from researcher to user, but can equally be a ‘pull’ from policymakers [3, 18], or an interactive relationship with influences operating in both directions [10, 19–21]. That is, translation processes can be understood as a system of complex, nonlinear interactions between researchers, evidence, context and decision makers [2, 20, 22]. Importantly, an interactive model recognizes that links and processes may be initiated by policymakers or researchers.

As a specialized public health research group, the Prevention Research Collaboration (PRC) at the Charles Perkins Centre and based at the University of Sydney is one such group that has been devoted to research translation for 20 years. PRC was established in 2004 with a mix of academic research grants and government funding to rapidly build an evidence-base to guide their decision-making on the emerging priority of child obesity. In addition to their established academic credentials, the key academic leaders had a history of collaborating with government on applied research, having worked with the state government on smaller funded research groups in the areas of nutrition and physical activity since the early 1990s. At this time evidence-based public health practice was emerging, but little attention had been given to aligning research activities with policy and strategic priorities. The stability of key government public health infrastructure and PRC’s commitment and skills in networking and knowledge brokerage enabled a high degree of trust, co-development, and co-authorship throughout an extended period of time. As a result, PRC could undertake a mix of policy-initiated and investigator-initiated research, focused on key policy areas in the prevention and control of chronic diseases (physical activity, nutrition, obesity prevention, tobacco control, epidemiology, and health promotion program evaluation).

The PRC infrastructure has included an academic Director, a policy-oriented Strategic Advisory Board, an Executive Officer/knowledge broker role and a center administrative manager. The PRC staff comprised 20–30 faculty positions, either on fixed term contracts or tenured appointments. The former were funded by research grants, while the latter were university-funded and carried both teaching and research responsibilities. All staff had expertise in public health research, spanning diverse methods and disciplines, enabling collaboration on policy-relevant research. Twenty years on, the research academic sector is now placing greater expectations on researchers to demonstrate ‘nontraditional’ research impacts [23, 24]. For example, the impact agenda, which emphasizes accountability for publicly funded research beyond academic merit to broader societal benefits, urges research that is collaboratively designed with consumers, policymakers or industry partners. Given the interest in translating evidence and research into population impact, the PRC represents a distinctive case study for examining characteristics and patterns of research–policy–practice partnerships.

In this paper, we profile the research work of this applied public health research organization, highlighting its scope of research and collaborative attributes. Specifically, this case study shows the mix of content and study types for both policy-initiated and investigator-initiated research and considers the extent to which this reflects PRC’s distinctive background as an applied research group.

## Methods

To undertake a descriptive analysis of PRC research, the authors systematically identified a recent set of research programs and projects for consideration. Firstly, the authors identified all journal articles published by PRC over the period 2018–24. PRC publications were those where the primary author or senior author was a member of PRC or there were two or more PRC authors. Those publications by a senior PRC author were confirmed with the relevant author as being produced within their role and remit as a PRC member. Secondly, to gain a more complete picture of the full range of PRC research, annual organizational reports, and funding specified workplans (herein referred to as PRC reports) for 2013–23 were examined to identify major research programs. The timeframes were determined by the availability of systematically documented data, PRC began formal tracking of publications in 2018 and maintained standardized workplans from 2013 to 2023 that aligned with specific funding arrangements. Records prior to these dates were incomplete due to organizational changes in physical premises, systems, and personnel. A major research program was defined as comprising one or more related projects, involved more than one PRC researcher, involved considerable financial and/or human resources, and was longer than 3 months in duration. There was no requirement that they had to be specifically investigator- or policy-led programs.

For each method of identifying PRC’s body of research, initial listings were reviewed (B.O.H.) and checked by other authors (L.K., P.P.). The two differing sources of identifying the body of research were undertaken to ensure that no major pieces of work, such as any that may not have produced peer-reviewed publications, were excluded. These methods also ensured that a comprehensive and representative set of research work was identified and used for analysis in this study.

Utilizing an adapted stages of evidence-building framework originally developed by Bauman and Nutbeam [8] and as depicted in Figure 1, the authors classified PRC’s journal publications and major research programs identified through PRC reports to a study type category. The additional category of ‘methodology development’ was introduced, to separately identify those projects which focused on measurement and development of research and evaluation methodologies. The topic of the research was also identified. Further, the journal publications and PRC report-derived research programs were classified into whether they were initiated by the PRC researcher (investigator-initiated) or were directed through, or in collaboration with, a policy/practitioner (policy-initiated). Policy organizations included government departments and agencies (such as the Ministry of Health), while practitioner organizations encompassed service delivery organizations (such as nongovernmental organizations and health services). These were combined into a single category because both represent research users who commission or collaborate on research to inform decisions and practice, distinguishing them from purely investigator-driven inquiry. For the journal publications, the author list was reviewed to determine whether authors from policy–practitioner organizations were included, which organizations were represented, and whether authors from other universities outside of the University of Sydney were included.

## Results

### Initiator of research

The mix of investigator-initiated and policy-initiated research differed substantially by data source (Table 1). Journal publications (2018–24) were predominantly investigator-initiated (86.7%, *n* = 189), while research projects identified from PRC reports (2013–23) showed the opposite pattern, with 60.0% (*n* = 102) being policy-initiated. This represented a significant 46.7% difference (95% CI 36.7%–54.7%) in the proportion of policy-initiated work between the two sources.

**Table 1:** Policy-initiated versus investigator-initiated research outputs classified by source of information

	Source			
	PRC journal articles 2018–24		PRC reports 2013–23	
Research instigator	*N*	%	*N*	%
Investigator-initiated	189	86.7	68	40.0
Policy-initiated	29	13.3	102	60.0

### PRC research profile across public health evidence-building typology

**Figure 2:**
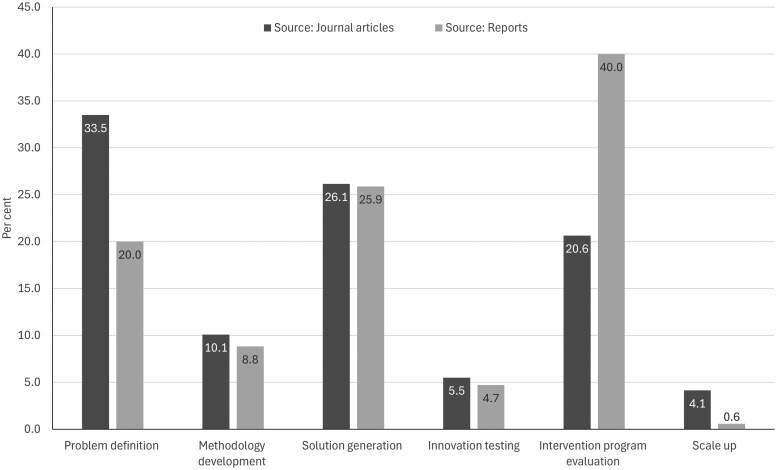
Evidence building study typology by PRC source of information

Figure 2 shows that the pattern of study types (in line with public health evidence building typology) differed according to whether the project/program was sourced from PRC reports or whether it was a journal article. The former were more likely to include intervention program evaluations and less likely to include projects that were designed to identify and articulate the nature, size and extent of a public health problem (i.e. problem definition), when compared to PRC journal articles.

### Investigator-initiated versus policy-initiated research programs and public health evidence typology

The distribution of PRC’s research portfolio demonstrates distinct patterns across evidence-building study types and the sources that were used to collect data (PRC journal publications versus PRC reports) (Table 2). Analysis of journal publications reveals that certain study types were exclusively investigator-initiated, specifically problem definition (*n* = 73, 100%) and innovation testing (*n* = 12, 100%). Similarly, methodology development (*n* = 20, 90.9%), solution generation (*n* = 53, 93.0%), and translation studies (*n* = 8, 88.9%) were predominantly investigator-initiated. The exception was intervention program evaluation, where there was a more balanced distribution between investigator-initiated (*n* = 23, 51.1%) and policy-initiated (*n* = 22, 0.5%) research.

**Table 2:** Investigator versus policy-initiated research programs by public health evidence typology

	Investigator-initiated		Policy-initiated	
Evidence building study type	*N*	%	*N*	%
Source: PRC journal publications				
Problem definition	73	100.0		
Methodology development	20	90.9	2	0.1
Solution generation	53	93.0	4	0.1
Innovation testing	12	100.0		
Intervention program evaluation	23	51.1	22	0.5
Translation	8	88.9	1	0.1
Source: PRC reports				
Problem definition	27	79.4	7	20.6
Methodology development	9	60.0	6	40.0
Solution generation	7	15.9	37	84.1
Innovation testing	2	25.0	6	75.0
Intervention program evaluation	22	32.4	46	67.6
Translation	1	100.0		

The pattern observed in PRC reports presents a different profile. Policy-initiated research dominated several categories, particularly solution generation (84.1%, *n* = 37), innovation testing (75.0%, *n* = 6), and intervention program evaluation (67.6%, *n* = 46). Conversely, problem definition remained primarily investigator-initiated (79.4%, *n* = 27), though not exclusively so as was the case in journal publications. Methodology development showed a more balanced distribution between investigator-initiated (60.0%, *n* = 9) and policy-initiated (40.0%, *n* = 6) work.

### Examples by study type

For the period 2013–23, 170 major research programs by our review of PRC reports were identified. During the period 2018–23, 218 PRC publications were identified. Examples of the types of research across the public health typology of evidence are provided in Table 3.

**Table 3:** Examples of major research projects by study type

	Examples	
	Investigator-initiated	Policy-initiated
Problem definition	Lancet Series on Physical Activity [25, 26] Trends in e-cigarettes use [27]	Schools Physical Activity and Nutrition survey [28]
Solution generation	The socio-ecological determinants of help-seeking practices and healthcare access among young men: a systematic review [29] Guide on workplace health promotion Evidence on sedentary behavior	Rapid review on sugar and health Rapid review on schools’ canteens Rapid review on voucher schemes to promote increased participation in sport and active recreation Impacts of outdoor gyms in public spaces [30]
Methodology development	Single item physical activity questionnaire for population surveillance Livability indicators for Local Government Areas Measurement of sitting—validation study/measure [31, 32]	Baseline survey development for Make Healthy Normal Social Marketing Campaign Technical advice on Government Healthy Eating and Active Living strategy online survey
Innovative testing	Case for citizen science in public health/prevention Sedentary behavior and health intervention testing	Healthy and Active for Life Program (HAL Online) RCT
Intervention program evaluation planning and assessing the implementation and evaluation of the intervention	Evaluation of the First Nations community intervention Evaluation of Your Brain Matters Demetia Risk Reduction Campaign [33] Using Facebook to recruit for public health campaign evaluation [34]	NSW Get Healthy Coaching and Information Service Evaluation [35] Make Healthy Normal Campaign Evaluation [36] NSW Quitline evaluation [37, 38] NSW Active Kids Vouchers Evaluation [39, 40] Healthy Food and Drinks in NSW Health Facilities Evaluation [41]

### Involvement of others

Across the journal articles, co-authorship with policymakers/practitioners was frequent amongst both investigator-initiated and policy-initiated papers (42.5% and 50.0%), respectively. The number of policy/practitioner authors included in any given publication ranged from one to eight co-authors. The co-authors represented a range of health and social service professionals from Australian state and jurisdictional Government agencies, and a range of Nongovernment health and charitable agencies.

It is also apparent that the authors within PRC collaborate with other university and research organizations. Across all journal articles, 77.0% included authors from other research organizations both nationally and internationally, with policy-initiated articles having a slightly higher proportion of instances where other university and research organizations were included as an author (84.4%) than investigator-initiated articles (75.8%).

### Topics of research

A substantial proportion of journal articles were published in relation to physical activity (40.4%), obesity prevention (14.2%), and smoking/tobacco/vaping (12.8%) (Table 4), whereas the projects obtained from the PRC reports comprised 42.4% obesity prevention; 18.8% related to physical activity and 15.9% related to nutrition.

**Table 4:** Topic of research from PRC journal articles

	Source			
	PRC journal publications		PRC reports	
Topic of research	*N*	%	*N*	%
Physical activity (including sitting/sedentary)	88	40.4	32	18.8
Obesity prevention	31	14.2	72	42.4
Smoking/Tobacco/Vaping	28	12.8	6	3.5
Nutrition/healthy eating	19	8.7	27	15.9
Social marketing	8	3.7	17	10
Health promotion	7	3.2	4	2.4
Scaling up	7	3.2		
Workplace related interventions	7	3.2	4	2.4
Knowledge translation	4	1.8		
Media (including mass media)	4	1.8	2	1.2
Social media	2	0.9		
Other	13	6.0	6	3.5

## Discussion

This paper provides a descriptive profile of a public health research center, examining its contribution to chronic disease prevention and health promotion research over a sustained period. Our analysis reveals an organization that undertook research that spanned a diverse range of policy-initiated and investigator-initiated projects across the public health evidence typology and on a range of chronic disease prevention issues. This body of research has included epidemiological studies, methodological studies, intervention evaluation and scaled up implementation and translational research. The mix suggests a genuine evidence-building approach, that is of value, as we know that policy agencies require information on intervention implementation as well as the traditional and ongoing need for information about current public health problems [12]. The data also suggest that foundational research activities (problem definition, methodology development) tend to be investigator-initiated, while implementation-focused activities show greater policy-initiated representation, reflecting both the funding arrangements of the PRC (from policy organizations) and the need to ensure that there are collaborative policy partnership arrangements to facilitate translational initiatives. The overall pattern of work conducted through PRC, positively compares to the other organizations that focus on public health research in Australia [42–46].

This study shows that engaging in policy or practice research is not incompatible with scientific rigor [47]. PRC’s portfolio of investigator-initiated and policy-initiated research has arguably contributed to both the public health relevance of the overall body of research and also supported the development of a strong tradition in publishing high quality research. This study does suggest that investigator-initiated research dominates journal publications across all study types, while the review of PRC reports demonstrated more policy-initiated work, particularly in the implementation and evaluation phases of research. This reflects both PRC’s funding models but also broader patterns in academic publishing [48, 49]. Problem analysis and descriptive studies, which are typically more straightforward to investigate and publish, dominate public health journals, while real-world interventions and translational research appear less frequently [42–46]. Much of PRC’s intervention research was identified in organizational workplans rather than journal publications, reflecting the well-documented challenges with this type of research, namely that it often requires more time to undertake and produce outputs [47] and may face methodological challenges that make publication more difficult.

The policy-initiated components of PRC research reflect more recent initiatives to strengthen research translation processes through dedicated funding for intermediary organizations that bridge research and policy [14, 47]. By extension, a stable and sustained organization, with consistent infrastructure, funding, and staffing that enables long-term partnerships with policymakers, facilitates an evidence-building approach that can contribute to policy and practice. Brownson *et al.* [18] depict this as a virtuous cycle, where individuals and organizations support increasing capacity for evidence-based public health.

The alignment of the PRC’s work with policy priorities represents another critical element in PRC’s way of working. The mix of content expertise and high technical skills in areas across PRC research address significant public health problems related to chronic diseases and, accordingly, long-standing international and Australian policy priorities [50, 51]. This is the case for investigator-initiated as well as policy-initiated research. PRC’s mix of research content, with more recent additions in the areas of implementation and scaling up, social media, vaping, and social connectedness, also reflects PRC’s capacity to be agile, flex, and adapt to emerging evidence and changing government priorities. The PRC focus accords with recent initiatives internationally and in Australia to promote alignment of research and policy/practice [16, 52], and the need to demonstrate such alignment [24].

Prevention Research Collaboration’s sustained collaborative approach is evident in the high degree of co-authorship with policy and practice professionals and groups. This is particularly important since 40% of the research programs in our study were intervention evaluations, where partnerships are essential for facilitating the applicability and application of findings in practice. This is a significant partnership strategy that requires shared aims, ongoing communication and engagement, knowledge brokerage and ideally a high degree of trust. PRC has had long experience in working in this way, applying a wide range of other translational strategies, such as those more recently described and promoted by various international research groups [53]. This degree of collaboration and responsiveness is a key, distinctive factor in PRC’s profile and history. It in turn reflects stability in government commitment and investment in evidence-based chronic disease prevention over the past 20 years and PRC’s core infrastructure, endowed with a depth of skill and experience to actively engage with policymakers and their requirements. There are undoubtedly a mix of other factors involved that could not be analyzed for this study, including the long history of collaboration and strength of professional networks crossing policy and research agencies.

While indicating strong alignment between research and policy priorities, this descriptive account of PRC research does not directly demonstrate actual policy or practice impact. It is recognized that whilst these are meaningful outcomes for any applied research group, such impacts are particularly complex to identify and trace without examining a variety of data sources including end-users, translational strategies, the research and evidence context, and anticipated timing of impacts. This process requires specific studies, often on a case-by-case basis. While such evidence may help understand how impacts can be achieved, the features of the PRC research described that can typically support impacts include: sustained partnerships, dual funding streams, organizational stability, and skills within the research group in applied evaluation, translation, and understanding of policy-making processes. Further exploration of research programs would be required to indicate cases of actual policy or practice impact. Similarly, further exploration of the portfolio of translational strategies used and the circumstances in which they lead to actual impacts is of interest. These are critical considerations in light of the changing academic landscape with increasing attention for researchers to provide evidence of policy and practice impacts beyond achieving traditional research metrics and rankings [24]. At the same time, the continuity of content and coherence of PRC’s research programs suggest an ongoing contribution to knowledge building and academic goals, and consistent with PRC aims and strategic goals. These questions can be potentially explored through case studies, and contribute to an understanding of how public health research can have meaningful results, which is an area of interest to both policy makers, research funders, and universities [52].

This study focuses primarily on the more recent period of PRC research rather than the full 20 years of work, due to incomplete documentation of earlier activities. However, several methodological strengths enhance confidence in our findings. The continuity of infrastructure, staff, and funding sources indicate that patterns are similar over time. Additionally, the use of two different structured methods for identifying research programs/projects also provides a check that the authors have described the scope of PRC’s policy-initiated and investigator-initiated research, provides differing views of the body of work, and thus strengthens the value of this analysis as a credible account of the overall body of PRC research. It is worth noting that during the 20-year period covered in this study, there were a number of public health and prevention funding initiatives in Australia and within the state of New South Wales [54, 55]. Another key aspect of this period was the strong emphasis on evidence-based decision-making and policy-relevant research, and the value of commissioning and funding such research.

In conclusion, this descriptive profile of PRC research illustrates that a relatively small public health research group can be both academically productive and actively collaborate with practitioners and policymakers, producing a mix of evidence-building study types and sustaining this approach over a 20-year period. The PRC profile demonstrates that having multiple funding sources that support both policy-initiated and research-initiated research programs, combined with stable, ongoing collaborations with policy agencies, internal focus on active engagement with policy processes, and methodological diversity, can foster policy relevance alongside academic rigor. These insights provide impetus for PRC to shift the balance more strongly towards translational research, as effective implementation is a critical endpoint for achieving public health benefit. They may also prove valuable for other research groups seeking to enhance their contributions to both knowledge generation and public health action in the field of chronic disease prevention.

## Data Availability

Deidentified data from this study are not available in a public archive. Deidentified data from this study will be made available (as allowable according to institutional IRB standards) by emailing the corresponding author.

## Artificial Intelligence

Artificial intelligence was not used in the development of this manuscript.
